# Layered risk factors for sudden unexpected infant death

**DOI:** 10.1186/s40621-026-00726-w

**Published:** 2026-10-08

**Authors:** Gina S. Lowell, Rojin Ahadi, Sumihiro Suzuki, Felicia Clark, Christie Lawrence, Meredith Reynolds, Kyran Quinlan

**Affiliations:** 1 Department of Pediatrics, Rush University Medical Center, 1645 W.Jackson Blvd, Suite 200, Chicago, IL 60612 USA; 2 Department of Preventive Medicine, Rush University Medical Center, Chicago, IL USA; 3 Department of Women, Children and Family Nursing, Rush University College of Nursing, Chicago, IL USA; 4 Cook County Medical Examiner’s Office, Chicago, IL USA

**Keywords:** SUID, Prevention, Social influences of health

## Abstract

**Background:**

Sudden Unexpected Infant Death occurs about once a week in Cook County, Illinois, the second largest county in the United States, where SUID disproportionately affects families from high hardship communities. The Cook County SUID Case Registry was established in 2019 to collect, analyze, and disseminate data to advance SUID prevention.

**Methods:**

Cook County SUID Case Registry data from 2019 to 2023 were analyzed for descriptive characteristics including infant age, race, ethnicity, gestational age, birth weight, maternal age, prenatal/household smoke exposure, and sleep environment hazards. Case reviews (including narrative review) identified patterns of risk which were quantified to provide proportions of SUID that occurred among families with histories of domestic violence, substance use, and among families whose infant died when they were away from their usual home (“temporary stays”). SUID that occurred among preterm infants during temporary stays were further analyzed to detail risk among this population.

**Results:**

From 2019 to 2023, 208 sleep-related infant deaths occurred in Cook County, Illinois. These deaths peaked at 1–2 months of age. The rate among non-Hispanic Black and Hispanic infants was 14 and 2.3 times that of white infants respectively. Most sleep-related infant deaths involved unsafe sleep situations including a sleep surface other than a crib/bassinet [175/208 (84%)], surface sharing [135/206 (66%)], and soft bedding in the sleep environment [190/205 (93%)]. Families whose infants died experienced social challenges including histories of domestic violence [32/182 (18%)], maternal substance use [53/185 (29%)] and temporary stays outside of the usual home [34/208 (16%)]. Preterm infants who died during temporary stays [12/34 (35%)] had multiple sleep environment hazards [non-supine sleep (4/12, 33%), non-approved sleep surface (12/12,100%), surface sharing (10/11, 91%), soft bedding (12/12,100%)] and social challenges [histories of domestic violence (3/12, 25%), maternal substance use (5/12, 42%) and child welfare involvement (9/12, 75%)].

**Conclusion:**

SUID in Cook County is closely tied to complex social issues and high hardship resulting in layered risk for vulnerable infants. Future work is needed to better understand how these challenges translate into unsafe sleep situations. Preventing SUID connected to these issues necessitates approaches that engage and equip families alongside trusted partners within communities most impacted by sleep-related infant death.

## Introduction

Sudden Unexpected Infant Death (SUID) is the leading cause of infant death once discharged home from the hospital after birth, and includes deaths from accidental suffocation, sudden infant death syndrome (SIDS), and unknown causes [1]. The majority of these deaths occur during sleep, and are also referred to as sleep-related infant deaths [2]. There has been little progress in further reduction of these deaths following the successful 1994 Back to Sleep campaign, which dramatically reduced the annual number of SUID from ~ 7500 to ~ 3500 [3]. Since that time, ample evidence regarding hazards in the sleep environment have guided the American Academy of Pediatrics (AAP) safe sleep recommendations, which are anchored in placing an infant to sleep on their back, on a firm and flat sleep surface, with no soft objects in the sleep environment. This guidance prevents accidental suffocation during sleep and reduces the risk of SUID from unknown causes. Additional evidence-based recommendations to reduce the risk of these deaths include room sharing without bed sharing, offering a pacifier for infant sleep, avoiding overheating, breastfeeding, attending regular prenatal care, avoiding prenatal and household tobacco smoke exposure, and avoiding alcohol, marijuana and illicit drug use during pregnancy and after birth [2, 4]. Adherence to these recommendations are significantly impacted by economic hardship, complex social circumstances, and access to trusted healthcare, driving persistent differences in SUID rates by race/ethnicity, geography, and socioeconomic status [5]. 

The triple risk model offers a conceptual framework that describes sudden infant death as occurring when an intrinsically vulnerable infant (e.g. physiologically, anatomically, or genetically predisposed to impaired arousal from sleep) is exposed to exogenous stressors (e.g. an asphyxiating environment) during a critical period of development (e.g. the first months of life) [6]. Modifiable sleep environment risk factors, including non-supine sleep, not sleeping on a firm and flat surface, surface sharing with adults or other children, and/or soft bedding in the sleep environment, are dominant characteristics of sleep-related infant deaths. In the most recent analysis of national data from the Center for Disease Control and Prevention’s (CDC) SUID Case Registry (SUID-CR), these factors were present in 98.5% of SUID with complete case information [7]. Furthermore, an analysis of SUID-CR data examining deaths on shared vs. non-shared sleep surfaces found at least 76% of all SUID had multiple unsafe sleep factors present [8]. What is less understood is how the interplay of economic hardship and complex social circumstances lead to unsafe sleep environments that ultimately contribute to or cause these infant deaths.

The SUID-CR currently funds 32 states and jurisdictions to collect, categorize, analyze, and disseminate their regions’ SUID data to inform prevention [9]. Cook County, IL joined the SUID-CR in 2019 as the second largest county in the US, where SUID occurs, on average, once a week, and disproportionately affects families from high hardship communities as defined by the Hardship Index, a composite score that takes into account layered stressors including poverty, unemployment, crowded housing, and education, and is highly correlated with other measures of economic hardship and poor health outcomes [10, 11]. The Cook County SUID-CR team reviews each SUID in partnership with the Cook County Medical Examiner’s Office (CCMEO) and Cook County’s Child Death Review teams (CDRT). These data are analyzed annually to quantify SUID rates, detail SUID characteristics, consider both immediate and upstream circumstances of each infant’s death, identify patterns and possible prevention insights, and share this information with families, community-based organizations, healthcare providers, and public health stakeholders to inform SUID prevention. The objective of this work was to use Cook County SUID-CR data to gain a better understanding of the social factors involved in these deaths to gain insights towards their prevention.

## Methods

We analyzed sleep-related infant deaths in the Cook County SUID-CR data from 2019 to 2023. Sleep-related deaths are defined by those that occurred during infant sleep. SUID that were not sleep-related (e.g. an infant witnessed to become unresponsive) were excluded from this analysis. Nearly all Cook County SUID are examined at the CCMEO, and data including autopsy reports, SUID investigation reporting forms (SUIDI-RF) [12], death scene investigations (DSI), child welfare investigations, police reports, and requested medical records are contained in the CCMEO’s LabLynx data system. The Cook County SUID-CR’s data coordinator accesses and synthesizes these data for the SUID-CR team, who reviews the contextual variables and narrative of each SUID prior to presenting the circumstances and suggested categorization to the CDRT for discussion and consensus regarding categorization.

Using the CDC’s SUID and SDY Case Registry algorithm [13], SUID may be categorized as: (1) Unexplained, no unsafe sleep factors, (2) Unexplained, with unsafe sleep factors, (3) Explained, suffocation with unsafe sleep factors, (4) Unexplained, possible suffocation with unsafe sleep factors, or (5) Incomplete case information. In order to categorize a SUID as “Explained, suffocation” there must be evidence of airway obstruction AND all of the following criteria are met: (1) There is a non-conflicting and reliable witnessed account; (2) There are no other potentially fatal finding(s) or concerning condition(s); (3) The infant’s age/developmental stage made suffocation feasible; (4) There is strong evidence of full external obstruction of both nose and mouth, or external neck or chest compression when found. If one or more of these criteria are not met despite evidence of airway obstruction, the SUID is categorized as “Unexplained, possible suffocation”. Suffocation mechanisms – or what caused the explained or possible suffocations – were captured through narrative review and scene reenactment photos (when depicted) and include soft bedding, overlay, wedging, and/or other. Unexplained, possible suffocation deaths are combined with Explained, suffocation deaths in Table 1 as these deaths had significant findings suggesting airway obstruction but did not satisfy one or more of the above criteria.

**Table 1:** Characteristics of sleep-related infant deaths in cook County, IL, 2019–2023. Percentages are reported for those sleep-related infant deaths for which the variable was known and are rounded to the nearest integer. Denominators less than 208 reflect the number of deaths in that category for which the variable was known

Sleep-related infant death characteristics (n=208)	Number (percent)
**Race/Ethnicity combined groups**	
• Non-Hispanic Black • Hispanic • Non-Hispanic White • Multiracial	153 (74%) 34 (16%) 19 (9%) 2 (1%)
**Infant vulnerability**	
• Preterm (< 37 weeks gestation) • Low birth weight (< 2500 g) • NICU stay	56/206 (27%) 54/199 (27%) 66/205 (32%)
**Categorization**	
• Explained, suffocation (40) and Unexplained, possible suffocation (16) • Unexplained, unsafe sleep factors • Unexplained, no unsafe sleep factors • Incomplete case information	56 (27%) 146 (70%) 1 (1%) 5 (2%)
**Sleep Environment**	
• Side or Stomach (found position)	88/203 (43%)
• Location other than crib/bassinet	175 (84%)
• Adult Bed	• 140/175 (80%)
• Couch/Futon	• 18/175 (10%)
• Inclined product	• 7/175 (4%)
• Other	• 10/175 (6%)
• Surface Sharing	135/206 (66%)
• Soft bedding	190/205 (93%)
**Crib available**	144/190 (76%)
• Crib available when placed to sleep in location other than crib/bassinet (*n* = 175)	116/157 (74%)
• Crib available when placed to sleep in location other than crib/bassinet during temporary stay (*n* = 29)	12/23 (48%)
**Social Environment**	
• Prenatal/Household Tobacco Smoke • Maternal History of Domestic Violence • Maternal History of Substance Use • Temporary Stay	30/167 (18%) 32/182 (18%) 53/185 (29%) 34 (16%)

Footnote: Data unknown for: Preterm (2), Low birth weight (9), NICU stay (3), Side or Stomach (found position) (5), Surface sharing (2), Soft bedding (3), Crib available (18), Crib available when placed to sleep in location other than crib/bassinet (18), Crib available when placed to sleep in location other than crib/bassinet during temporary stay (6), Prenatal/Household Tobacco Smoke (41), Maternal History of Domestic Violence (26), Maternal History of Substance Use (23)

The data coordinator enters the required variables and categorization for each SUID into the Pediatric National Fatality Review-Case Reporting System (Pediatric NFR-CRS). The SUID-CR team provides quality assurance of data entry, and quality control regarding categorization is provided by the CDC and the National Center for Fatality Review and Prevention’s (NCFRP) Technical Assistance teams who provide review and discussion of data entry and categorization disagreements quarterly with the SUID-CR team. Any changes to categorization after quarterly review are entered into the Pediatric NFR-CRS.

SUID that are not examined at the CCMEO are ascertained through a data reconciliation process with IL Vital Records when annual birth/death cohorts are closed. IL Vital Records provides linked birth/death certificate data for those infants whose death certificates have been assigned an ICD-10 code of R95, R99, W75, W76, W76-W84, and Y33-Y34 and includes date of birth, date of death, sex, race/ethnicity as reported on the death certificate, county and zip code of residence, county of occurrence, mother’s age at infant’s birth, gestational age at birth, cause of death, manner of death, ICD-10 code, and description of injury when death occurred. The SUID-CR data coordinator crosschecks SUID-CR data with IL Vital Records data and enters those infants not examined at the CCMEO into the Pediatric NFR-CRS; data for these infants are limited to the listed birth/death certificate data elements provided. Nine (4%) Cook County SUID were ascertained in this manner.

Descriptive characteristics including infant age, race/ethnicity combined groups (non-Hispanic Black (NHB), Hispanic, non-Hispanic White (NHW), other), gestational age, birth weight, neonatal intensive care unit (NICU) stay, maternal age, prenatal/household smoke exposure, sleep environment hazards, and categorization were analyzed. Corrected gestational age was calculated by subtracting the number of weeks the infant was born early (before 40 weeks) from their chronological age in weeks at death. Sleep-related infant death rates were calculated per 100,000 Cook County resident live births and preterm live births as provided by the Illinois Department of Public Health Division of Vital Statistics.

Details of multiple social factors involved in these deaths are captured in the SUID-CR data as well as the case narratives. Economic hardship was assessed by the infant’s health insurance status, with Medicaid coverage indicating family income below 213–318% of the federal poverty level; this variable was available for 87% of case reviews. The presence of domestic violence, substance use and issues related to housing and caregiver supervision was determined according to the Pediatric NFR-CRS Data Dictionary [14]. Exposure to domestic violence was defined as actual or threatened physical or sexual violence or psychological and emotional abuse directed toward the infant’s mother, and could also include domestic disturbance complaints to which law enforcement responded. The history of substance use was defined by whether the infant’s mother used drugs or substances during pregnancy (including cocaine, heroin, marijuana, methamphetamine, alcohol, opioids, and/or medications used to treat drug addiction), whether the infant was born substance exposed, whether the infant had neonatal abstinence syndrome, and/or whether the mother had a substance use history. Housing instability was defined as a housing challenge such as inability to pay rent or mortgage payment, overcrowding, moving frequently, eviction, staying with relatives or friends, or spending the bulk of their income on housing. Child welfare involvement was defined as any parent or caregiver of the infant having had child welfare services as a child or adult. Information regarding these variables were obtained from review of medical records (prenatal, birth hospital, and/or pediatric clinical care documentation), child welfare records (when a child welfare investigation was completed), police investigation reports, and/or the SUIDI-RF. Of these data sources, the SUIDI-RF is the only standardized form used by the CCMEO death scene investigators and was available for 75% of case reviews. Information regarding domestic violence and maternal substance use was available from 88% to 89% of case reviews, respectively. Case reviews (including narrative review) identified patterns of risk which were quantified to provide proportions of SUID that occurred among families with histories of domestic violence or substance use; and among families whose infant died when they were away from their usual home, or during “temporary stays”. SUID that occurred among preterm infants during temporary stays were further analyzed to detail risk among this population. Percentages are reported for those sleep-related infant deaths for which the variable was known.

## Results

**Fig. 1:**
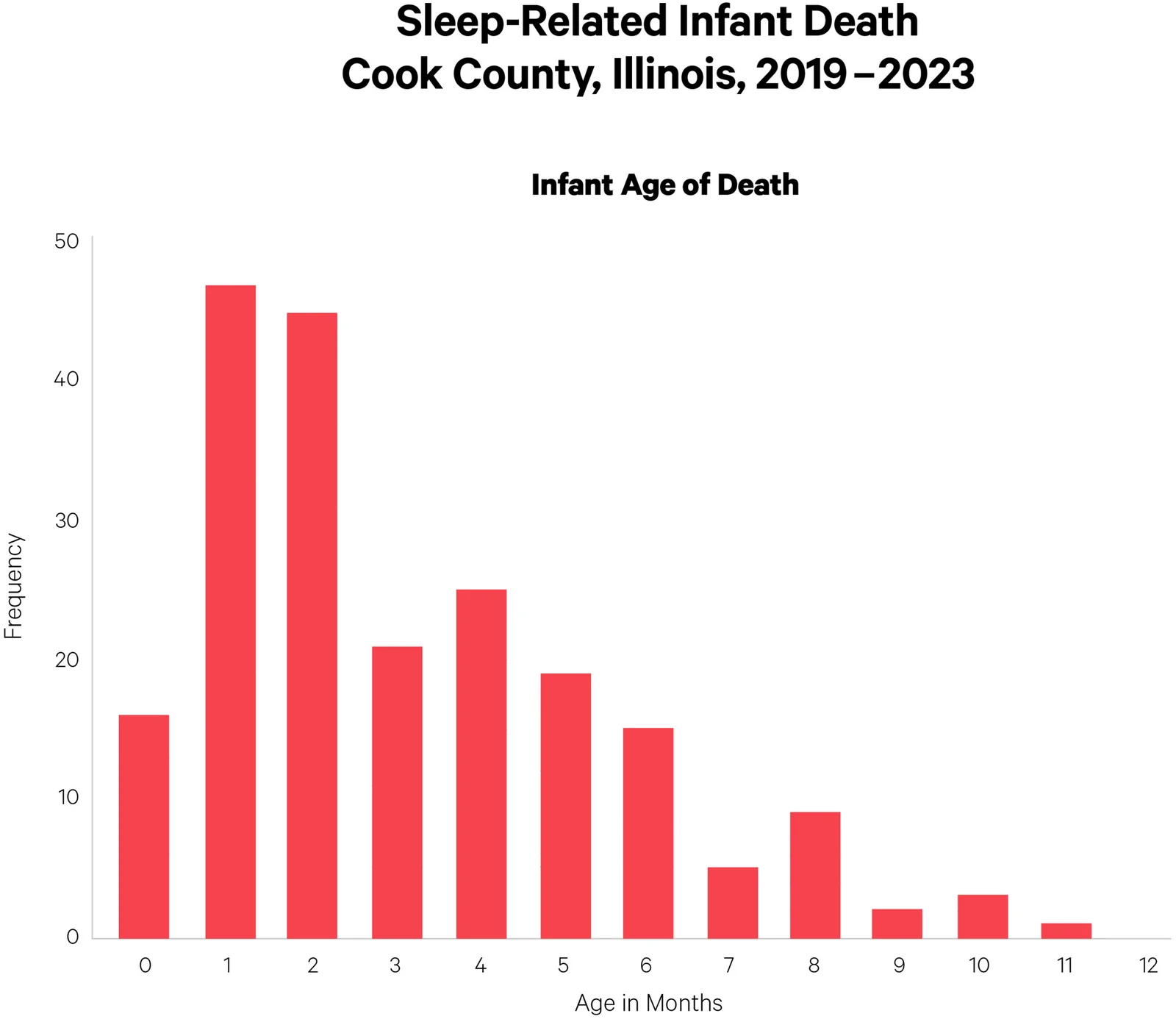
Age Histogram of sleep-related infant deaths (208), Cook County, IL, 2019–2023. Source: Cook County SUID Case Registry

**Fig. 2:**
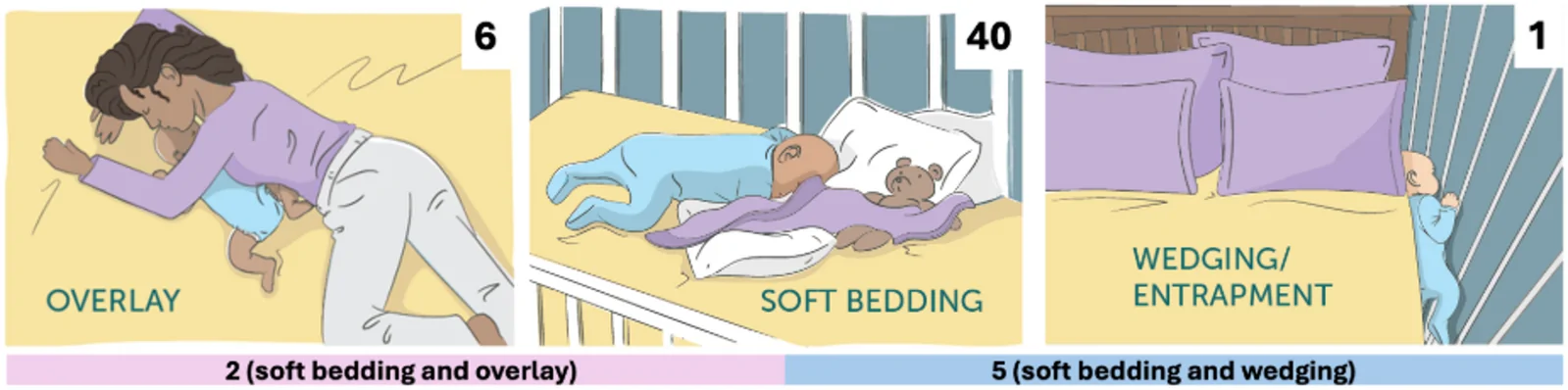
Mechanisms of Airway Obstruction for suffocation/possible suffocation-related SUID (*n* = 56). Two infants suffocated from mechanisms other than those pictured. Most infants (50, 89%) suffocated in a location other than a crib or bassinet. (Adult beds (42), Couches (4), Other (4)). Source: Cook County SUID Case Registry. Diagrams adapted From: Eunice Kennedy Shriver National Institute of Child Health and Human Development, NIH, DHHS. Safe to Sleep: Definitions and Terms. https://safetosleep.nichd.nih.gov/about/terms

From 2019 to 2023 there were 222 SUID, of which 208 (94%) were sleep-related and are the focus of this analysis. Sleep-related deaths peaked among infants aged 1-2-months old, and 85% of deaths occurred among infants aged < 6-months old. [Figure 1] Fifty-six (27%) sleep-related infant deaths were categorized as explained, suffocation (40) or unexplained, possible suffocation (16). Airway obstruction was attributed to soft bedding (47, 84%), overlay (8, 14%), wedging (6, 11%) and other mechanisms (2, 4%) among these 56 infants (mechanisms not mutually exclusive). [Figure 2] Race/ethnicity, infant vulnerability factors (preterm birth, low birthweight, requiring neonatal intensive care after birth), categorization, sleep environment factors (sleep position, location, surface sharing, and presence of soft bedding), and social environment characteristics of these deaths are detailed in Table 1. Most infants had public health insurance [166/181 (92%); 27 unknown].

Rates of sleep-related infant death for NHB infants (246/100,000), Hispanic infants (42/100,000) and NHW infants (18/100,000) were such that the rate ratios for NHB:NHW infants was 13.7:1, and for Hispanic:NHW infants was 2.3:1. **[**Table 2**]** The rate of sleep-related infant death for preterm Black infants was 545/100,000, meaning 1 in 183 preterm Black infants died during this time. **[**Table 3**]**

**Table 2:** Sleep-related infant death rates and rate ratios by race/ethnicity combined groups

Cook County, IL 2019–2023	Non-Hispanic black	Hispanic	Non-Hispanic white
Sleep-Related Infant Death (n)	153	34	19
Resident Live Births	62,259	80,846	103,528
Rate (n/100,000 resident live births)	246	42	18
Rate Ratio (as compared to NHW)	13.7	2.3	Referent

Footnote: Sources: Sleep-Related Infant Deaths (208) are from the Cook County SUID Case Registry. Two infants were multiracial and not included in these rate calculations. Cook County Resident Live Births were obtained from the Illinois Department of Public Health’s Division of Vital Statistics. (https://dph.illinois.gov/data-statistics/vital-statistics.html)

**Table 3:** Preterm sleep-related infant death rates and rate ratios by race/ethnicity combined groups

Cook County, IL 2019–2023	Non-Hispanic Black	Hispanic	Non-Hispanic White
Sleep-Related Infant Death, preterm infants (n)	50	3	3
Preterm Resident Live Births	9,177	8,413	8,755
Preterm Rate (n/100,000 preterm resident live births)	545	36	34
Rate Ratio (as compared to NHW)	16.0	1.1	Referent

Footnote: Sources: Preterm Sleep-Related Infant Deaths (56) are from the Cook County SUID Case Registry. Cook County Preterm Resident Live Births were obtained from the Illinois Department of Public Health’s Division of Vital Statistics. (https://dph.illinois.gov/data-statistics/vital-statistics.html)

Sleep-related deaths occurred among 32/182 (18%) families with a documented history of domestic violence and 53/185 (29%) families with a documented history of maternal substance use. Sleep-related deaths occurred among 34 (16%) infants during temporary stays, 12 (35%) of which occurred among preterm infants. These 12 infants had a corrected gestational age of < 5-months at their death, 10 (83%) had NICU care and 4 (33%) infants were one of twins. Three (25%) deaths were categorized as accidental suffocation. No crib was available in the temporary stay location for 5 of 10 (50%) infants for whom this information was known, and six infants (50%) were in the care of non-parent supervisors when they died. Multiple unsafe sleep factors (non-supine sleep [4/12, 33%], non-approved sleep surface [12/12,100%], shared sleep surface [10/11, 92%], and soft bedding [12/12,100%]) were present. Surface sharing occurred on adult beds (9/10, 90%); with adults (9/10, 90%), siblings (8/10, 80%), or both adults and siblings (6/10, 60%). Five (42%) families had a history of maternal substance use, 3 (25%) families had a history of domestic violence, and 9 (75%) families had a history of child welfare involvement.

Narrative review of the circumstances of these 12 preterm infant deaths did not always reveal the precise reason for temporary stays but often involved housing instability and reliance on multiple caregivers (especially grandparents) for childcare of both the infant and siblings due to parent employment and/or social circumstances. Parents frequently had histories of child welfare involvement when they themselves were children, and sometimes as parents. Both parents and caregivers disclosed ongoing situations of domestic violence. Although substance use histories were prevalent, impairment at the time of infant death was infrequent.

## Discussion

A significant proportion of SUID in Cook County involves complex social risks layered on economic hardship and infant vulnerability which challenge prevention. Preterm Black infants had the highest subgroup rate of sleep-related infant death, such that 1 in 183 preterm Black infants in Cook County died during this 5-year period. Among preterm infants who died during temporary stays, multiple sleep environment hazards and socioeconomic risks were prevalent. Detailing the characteristics of sleep-related infant deaths among families facing significant hardship begins to shed light on how social circumstances present barriers to safe infant sleep practices.

Infant vulnerability such as prematurity, low birthweight or prenatal smoking, and sleep environment risks such as bedsharing, are typically assessed individually [15] and sometimes in combination [16], conferring various levels of elevated risk for exposed infants. High social vulnerability – a multidimensional framework reflecting financial hardship, unstable employment, low educational opportunity, housing instability, transience and frequent moves, chronic health conditions, domestic violence, and child welfare involvement – has been shown to correlate with a higher cumulative total of risk factors for sleep-related infant deaths [17]. Understanding how social vulnerability operates to layer risk in the infant’s sleep environment is key to advancing approaches to preventing these deaths.

Economic hardship, housing instability, domestic violence, substance use, child welfare involvement, chronic health conditions and mental health difficulties can be challenging to detangle to understand the magnitude of risk each exerts on sleep-related infant death [18]. Barrett et al. conducted in-depth, semi-structured interviews of families from northeast England involved with child welfare services and found difficulties such as having to leave one’s home due to domestic violence disrupted infant sleep routines, necessitating the adoption of unsafe sleep practices. Parents also described how overwhelming anxiety drove desires and decisions to bedshare. The study authors suggest moving beyond simple “messaging” and information giving, instead opening meaningful conversation about parent capabilities and motivations set in a wider context of infant care practices and expectations [19]. 

Bedsharing occurred among 67% of Cook County infants who died during this time. When compared to a similar study of Cook County infants from 2015 to 2016 who died from undetermined causes only, 58% were co-sleeping when this variable was known [20]. When compared to infants from the Chicago Infant Mortality study [21], a case control study examining risk factors of infants who died of SIDS compared with infants who died from other causes, a lower proportion − 50.4% - of SIDS cases (and 30.4% of matched controls) were bedsharing at last sleep. While the study methods for these studies as compared to our study differ in their criteria, they quantified slightly lower proportions of infant deaths that occurred in a bedsharing or co-sleeping environment [20, 21]. 

Illinois Pregnancy Risk Assessment Monitoring System (PRAMS) data from 2016–2020 showed that when asked, “In the past 2 weeks, how often has your new baby slept alone in his or her own crib or bed?”, African American mothers were least likely to respond “always” (42.1% (38.5–45.7)) [22]. While the PRAMS questions do not fully capture bedsharing, this variable is taken to mean “no bedsharing”. A 2022 Voices of Child Health study asked a comprehensive community-based sample of Chicago parents about their usual infant sleep practices and found 37% reported placing infants to sleep in adult beds (but also did not fully capture bedsharing) [23]. 

In our local experience and efforts to prevent sleep-related infant deaths among Cook County’s most impacted communities [10], families have shared numerous barriers to practicing safe infant sleep, including substandard housing, having to share housing with other families, or housing instability; safety concerns from household or neighborhood violence; and economic pressures – getting up for work or school, alternating caregiving or relying on others for childcare to maintain employment, and a desire for closeness and bonding when parents return home – which may drive decisions to bedshare. Some of these concerns are echoed in focus groups and interviews of African American mothers from Washington DC that found low-income mothers often bedshared with their infants due to space constraints, to protect their infants from environmental hazards such as vermin and threatening hazards in the neighborhood, and to promote comfort and bonding [24]. 

The complexity of social factors involved in sleep-related infant deaths may play a part in the lack of recent progress in prevention, and addressing these issues is likely required to protect infants from these deaths. Nearly one-third of infants who died in Cook County had a history of requiring NICU care delivery, which has been associated with social inequities that contribute to multiple adverse short and long-term infant health outcomes (including infant mortality) as well as outcomes that are associated with sleep-related infant death (e.g. preterm birth, low birthweight, neonatal opioid withdrawal syndrome, lower breastfeeding initiation, and low clinic follow-up attendance) [25]. Recognizing NICU stays as a marker of layered risk for sleep-related infant death allows both neonatal teams and pediatric providers to utilize this as an opportunity for engaging in conversations with families that explores and addresses their barriers to safe sleep in the context of unmet social needs, which may serve to reduce the risk of and/or prevent these deaths.

In our local experience, when holding conversations with families from communities most impacted by sleep-related infant death, we have found that sharing our local data matters. Families and those who support them connect with and can provide insight into what is happening in their own communities. We have found that sharing the perspective of a parent who has lost her own infant in this way resonates strongly among families. We have also found that including two key elements in conversation – the frequency with which this happens in our communities (about once a week), and counseling to prevent accidental suffocation – deepens dialogue and anchors families in actionable steps they can take to prevent these deaths. Counseling to prevent suffocation is likely to reduce the risk of all sleep-related infant death as risk factors for both suffocation-related and unexplained SUID are highly similar [15]. Furthermore, African American mothers of newborns from an urban hospital setting were more likely to believe that unsafe sleep factors could lead to accidental suffocation (as compared to SIDS), and had greater confidence that their actions could protect their infants from accidental suffocation (as compared to SIDS) [26]. 

Limitations of our study include variability in death investigations and completeness of data collection. Most sleep-related infant deaths receive a death scene investigation (DSI) by a trained medicolegal death investigator and often include a scene reenactment using a doll in place of the infant to demonstrate the infant’s placed and found positions. A DSI offers the greatest opportunity for collecting complete information regarding the circumstances of the infant’s death, but families may refuse a DSI and/or doll reenactment. DSI relies on caregiver report regarding the circumstances surrounding the infant’s death, and is obtained as soon as possible after an infant’s death, when shock and grief are overwhelming. The limitation of self-report in the face of emotional distress is mitigated by reviewing multiple other sources who have documented the narrative at the time of their interaction with the infant’s caregiver(s) which is typically more proximal to the time of death. These include paramedics, police, and emergency room or ICU nurses and physicians. Review of these records typically enriches the narrative and can identify consistencies or discrepancies in the caregiver’s narrative.

The CCMEO death scene investigators complete an investigation report and are strongly encouraged to complete the SUIDI-RF, a standardized, highly detailed, 12-page form that maps onto the CDC and Pediatric NFR-CRS priority variables for SUID. The SUIDI-RF was not available for 25% of Cook County’s SUID, and SUIDI-RF that were available were not always fully completed, contributing to limitations in data completeness. Furthermore, ascertainment of variables such as domestic violence and substance use relies on caregiver self-report. These limitations are mitigated by the fact that many but not all of Cook County’s infant deaths also receive a child welfare investigation, which includes scene photos and reenactments, as well as detailed information about the social circumstances families face, and complete information on past child welfare involvement. Such in-depth interviews help to reveal the linkage of upstream factors that may have contributed to the infant’s death. That a subset of Cook County SUID receive a child welfare investigation contributes to ascertainment bias for those variables less often captured by death scene investigators.

Further limitations include the relatively small number of deaths during temporary stays which may not accurately represent that group as a whole. Finally, this analysis lacks a control group of surviving infants which would allow comparison of the rates of social factors between the two groups. Despite these limitations, characterizing the populations and geographies at greatest risk, alongside the sleep environment circumstances and other patterns has enabled tailored prevention approaches for our prevention team to implement, such as partnering with shelters to ensure cribs are available for infants in their care, reaching parent education groups and providing staff trainings for home visiting, doula and WIC agencies, and partnering with domestic violence programs to raise awareness of and promote safe sleep practices.

## Conclusions

In Cook County, SUID occurred among families with multiple complex social factors such as domestic violence, substance use and temporary housing. Further progress in SUID prevention will necessarily involve going beyond simple education on safe sleep to address unmet social needs that appear to be in the way of families with many social challenges routinely practicing all recommendations regarding safe infant sleep. The SUID-CR allows for greater understanding of the circumstances surrounding these deaths, the populations and geographies at risk, and permits pattern-finding that allows for tailored prevention approaches in partnership with communities with layered risks for sleep-related infant death. 

## Data Availability

The datasets generated and/or analyzed during the current study are not publicly available due to the National Center for Fatality Review and Prevention’s data access limitations but are available from the corresponding author on reasonable request.

## Declarations

### About this supplement

This article has been published as part of Injury Epidemiology Volume 13 Supplement 1, 2026: Proceedings of the 30th Annual Injury Free Coalition for Kids^®^ Conference: Forging New Frontiers: Injury Prevention in Evolving Environments. The full contents of the supplement are available online at https://link.springer.com/journal/40621/volumes-and-issues/13-1/supplement.

## Abbreviations

AAP: American academy of pediatrics
CDC: Centers for disease control and prevention
CCMEO: Cook county medical examiner’s office
CDRT: Child death review team
DSI: Death scene investigation
NCFRP: National center for fatality review and prevention
NFR-CRS: National fatality review case reporting system
NICU: Neonatal intensive care unit
PRAMS: Pregnancy risk assessment monitoring system
SDY: Sudden death in the young
SIDS: Sudden infant death syndrome
SUID: Sudden unexpected infant death
SUID-CR: Sudden unexpected infant death case registry
SUIDI-RF: Sudden unexpected infant death investigation reporting form
NHB: Non-hispanic black
NHW: Non-hispanic white
WIC: Women infants and children
